# Supplementary material for: The origins of haplotype 58 (H58) Salmonella enterica serovar Typhi
Source: Commun Biol. 2024 Jun 28;7:775. doi: 10.1038/s42003-024-06451-8 (PMC11213900; doi:10.1038/s42003-024-06451-8)
Supplement: Supplementary file 1 — Supplementary Information [file 42003_2024_6451_MOESM1_ESM.pdf]

Supplemental Figure 1: The phylogenetic structure of early H58 organisms and nearest neighbours.

Maximum likelihood rooted phylogenetic tree showing *S*. Typhi organisms (genotype 4.3.1) and nearest neighbours (genotypes 4.1 and 4.2) from our historical collection and from published literature (n=422 total). Genotype is indicated by branch colour, presence of QRDR mutation(s) are indicated by coloured circles at the end of the branches, and country of origin, presence of MDR, AMR mutations, and presence of incH1 plasmid are indicated by bars to the right of the tree and coloured as per the inset legend.

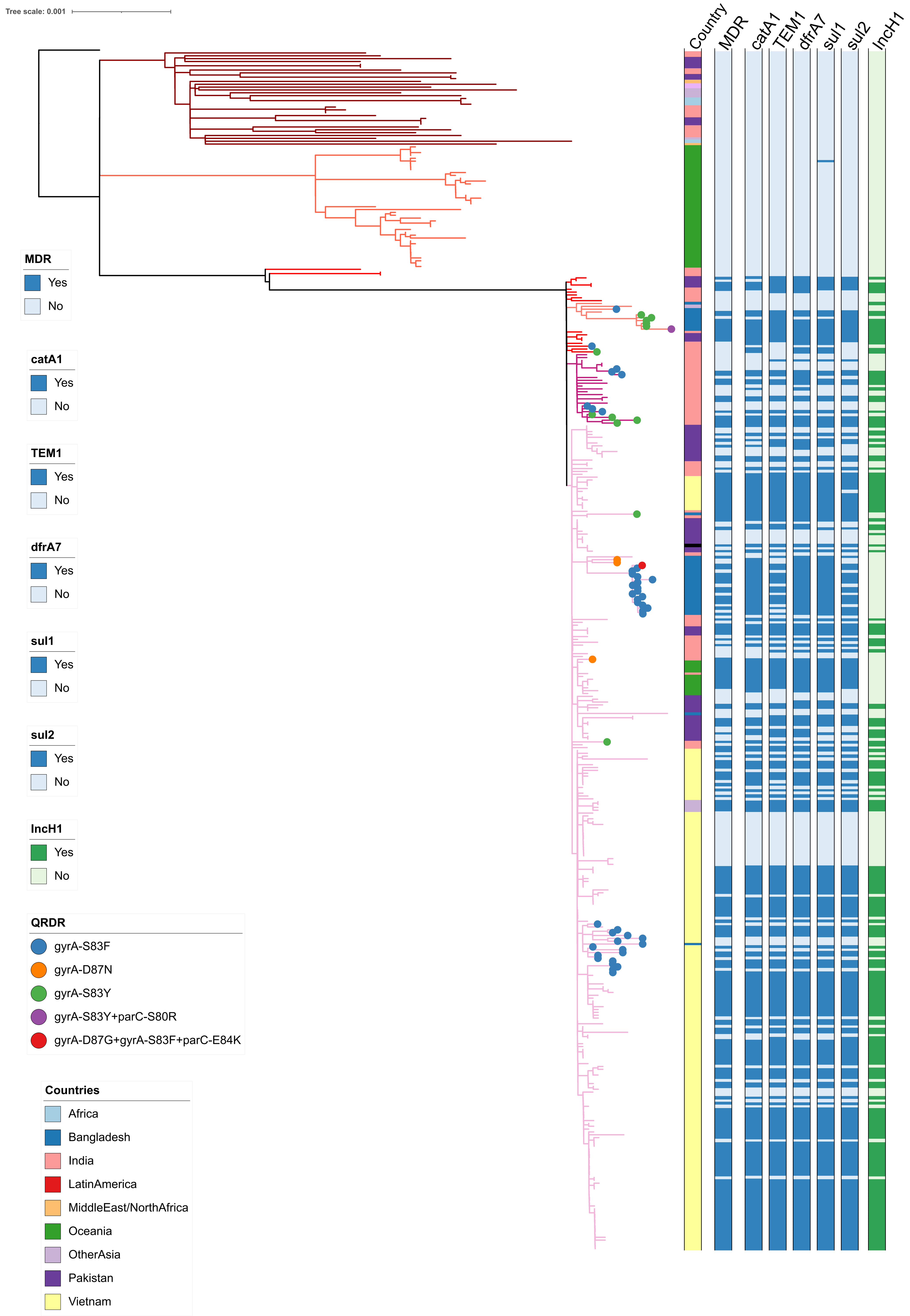
Tree scale: 0.001
Country
MDR
catA1
TEM1
dfrA7
sul1
sul2
IncH1
MDR
Yes
No
catA1
Yes
No
TEM1
Yes
No
dfrA7
Yes
No
sul1
Yes
No
sul2
Yes
No
IncH1
Yes
No
QRDR
gyrA-S83F
gyrA-D87N
gyrA-S83Y
gyrA-S83Y+parC-S80R
gyrA-D87G+gyrA-S83F+parC-E84K
Countries
Africa
Bangladesh
India
LatinAmerica
MiddleEast/NorthAfrica
Oceania
OtherAsia
Pakistan
Vietnam

Supplemental Figure 2: A genetic comparison of IncH1 plasmid content of early H58 *S.* Typhi isolates.

Zoomed in view of maximum likelihood phylogenetic tree including historical and published H58 isolates (n=206). Country of origin is indicated by the coloured circles at the tips of the tree. Gene content of an almost identical IncH1 plasmid is indicated by the colour to the right of the tree.

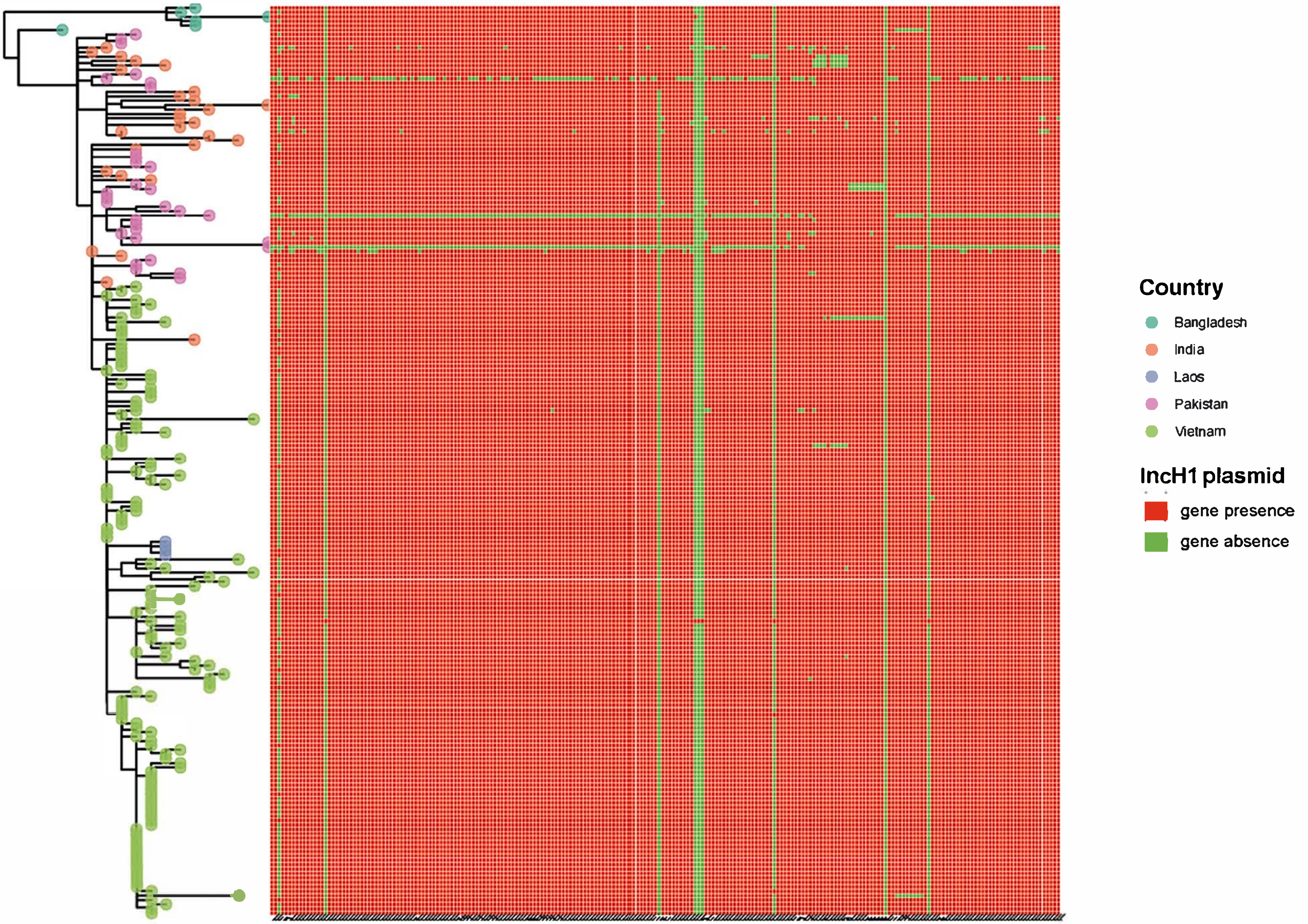
Country
Bangladesh
India
Laos
Pakistan
Vietnam
IncH1 plasmid
gene presence
gene absence

Supplemental Figure 3: An estimation of temporal signal in H58 *S*. Typhi data.

Path-O-Gen/TempEst results (analysis included n=345 isolates using an alignment of 724 non-recombinant SNPs), demonstrating strong correlation between sampling dates and root-to-tip distance (R2 = 0.4743).

☑ Best-fitting root

Function: heuristic residual mean squared

Dated Tips	
Date range	17
Slope (rate)	1.7153E-4
X-Intercept (TMRCA)	1949.1321
Correlation Coefficient	0.6887
R squared	0.4743
Residual Mean Squared	2.0781E-7

Sample Dates | Tree | Root-to-tip | Residuals | Node density

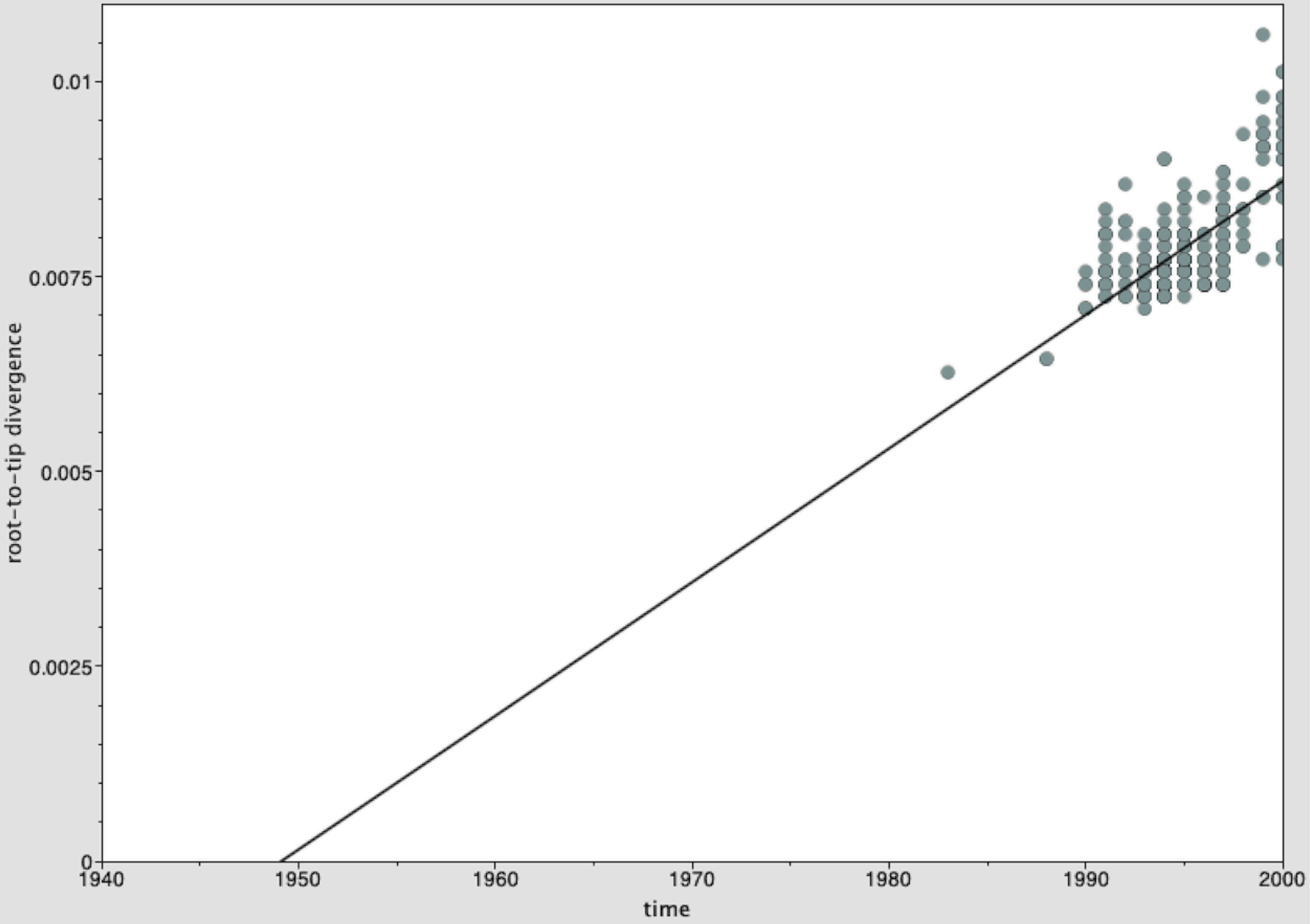

☐ Show ancestor traces

Tree loaded, 345 taxa, dated tips with range 17

Supplemental Figure 4: A TipDatingBeast estimation of temporal signal in H58 *S.* Typhi data.

TipDatingBeast results (analysis incorporated n=345 isolates and an alignment of 724 non-recombinant SNPs), demonstrating no overlap between the original mean rates of mutation and mean rates of date randomization.

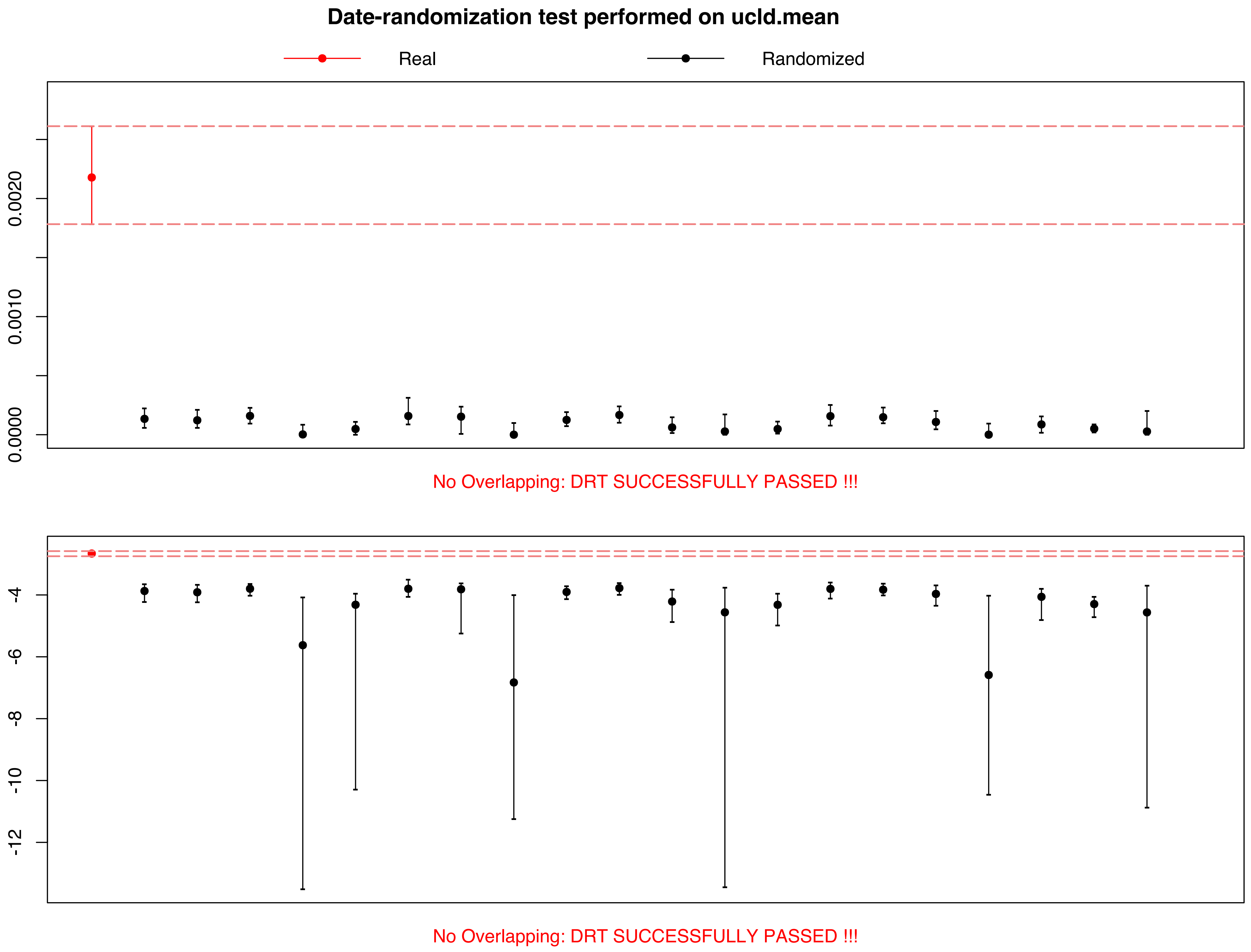
Date-randomization test performed on ucld.mean
Real
Randomized
0.0000
0.0010
0.0020
No Overlapping: DRT SUCCESSFULLY PASSED !!!
-4
-6
-8
-10
-12
No Overlapping: DRT SUCCESSFULLY PASSED !!!
