# Supplementary material for: The origins of haplotype 58 (H58) Salmonella enterica serovar Typhi
Source: Commun Biol. 2024 Jun 28;7:775. doi: 10.1038/s42003-024-06451-8 (PMC11213900; doi:10.1038/s42003-024-06451-8)
Supplement: Supplementary file 2 — Description of Additional Supplementary Files [file 42003_2024_6451_MOESM2_ESM.pdf]

Description of Additional Supplementary Files

File name: Supplementary Data 1
Description: Organism-level data and metadata for historical UKHSA S. Typhi isolates.

File name: Supplementary Data 2
Description: Organism-level data and metadata for published H58 and nearest neighbours S. Typhi isolates.
