# Supplementary material for: The origins of haplotype 58 (H58) Salmonella enterica serovar Typhi
Source: Commun Biol. 2024 Jun 28;7:775. doi: 10.1038/s42003-024-06451-8 (PMC11213900; doi:10.1038/s42003-024-06451-8)
Supplement: Supplementary file 4 — Reporting summary [file 42003_2024_6451_MOESM4_ESM.pdf]

nature portfolio

Corresponding author(s): Double-anonymous peer review submissions: write DAPR and your manuscript number here instead of author names.

Last updated by author(s): YYYY-MM-DD

Reporting Summary

Nature Portfolio wishes to improve the reproducibility of the work that we publish. This form provides structure for consistency and transparency in reporting. For further information on Nature Portfolio policies, see our Editorial Policies and the Editorial Policy Checklist.

Statistics

For all statistical analyses, confirm that the following items are present in the figure legend, table legend, main text, or Methods section.

n/a	Confirmed	
☐	☒	The exact sample size (*n*) for each experimental group/condition, given as a discrete number and unit of measurement
☒	☐	A statement on whether measurements were taken from distinct samples or whether the same sample was measured repeatedly
☒	☐	The statistical test(s) used AND whether they are one- or two-sided<br>*Only common tests should be described solely by name; describe more complex techniques in the Methods section.*
☒	☐	A description of all covariates tested
☒	☐	A description of any assumptions or corrections, such as tests of normality and adjustment for multiple comparisons
☒	☐	A full description of the statistical parameters including central tendency (e.g. means) or other basic estimates (e.g. regression coefficient) AND variation (e.g. standard deviation) or associated estimates of uncertainty (e.g. confidence intervals)
☒	☐	For null hypothesis testing, the test statistic (e.g. *F*, *t*, *r*) with confidence intervals, effect sizes, degrees of freedom and *P* value noted<br>*Give P values as exact values whenever suitable.*
☒	☐	For Bayesian analysis, information on the choice of priors and Markov chain Monte Carlo settings
☒	☐	For hierarchical and complex designs, identification of the appropriate level for tests and full reporting of outcomes
☒	☐	Estimates of effect sizes (e.g. Cohen's *d*, Pearson's *r*), indicating how they were calculated

Our web collection on statistics for biologists contains articles on many of the points above.

Software and code

Policy information about availability of computer code

Data collection

Data from United Kingdom Health Security Agency (UKHSA, formerly Public Health England) containing information on archived S. Typhi organisms isolated between 1980 and 1995 from travellers returning to the UK from overseas and receiving a blood culture were analysed.

The database was queried and organisms were selected from the following three categories: i) 126 S. Typhi with the E1 Phage type, which was thought to be associated with H58 [12], originating from South Asia (India, Nepal, Pakistan, and Bangladesh), ii) 159 S. Typhi organisms with a variety of non-E1 phage types originating from South Asia, and iii) 184 S. Typhi organisms with a variety of phage types (both E1 and non-E1) originating from locations outside of South Asia. A total of 470 S. Typhi organisms (out of 3,751 total S. Typhi isolated from returning travellers by UKHSA between 1980 and 1995) meeting these criteria were randomly selected, revived, subjected to DNA extraction and whole genome sequenced. Ultimately, our dataset was composed of 463 novel sequences generated as a component of this study and 305 existing sequences known to belong to the H58 lineage and its nearest neighbours, yielding a total of 768 whole genome sequences on which to structure subsequent analysis.

Data analysis

FastQC and FASTX-Toolkit bioinformatics pipelines were used to check the quality of raw reads [60, 61]. Six samples were excluded from the analysis, one was determined to not be Salmonella, one appeared to be comprised of multiple genotypes, and four samples were on a long branch length and were determined to be contaminated. Paired end reads for the remaining 464 samples were mapped to the S. Typhi CT18 reference genome (accession number: AL513382) [62] using the RedDog mapping pipeline (v1beta.10b, available at http://githib.com/katholt/reddog). RedDog uses Bowtie2 v2.2.9 [63] to map all raw reads to the CT18 reference genome and then uses SAMtools v1.3.1 [64] to identify high quality SNP calls. SNPs that did not meet predefined criteria (a minimal phred quality score of 30 and depth coverage of 5 were filtered out) [65]. A failed mapping sequence was defined as when <50% of total reads mapped to the reference genome. 2 isolates were excluded from additional analysis after mapping failed, due to depth coverage of less than 10 (as per the RedDog pipeline default). A concatenation of core SNPs that were present in >95% of all genomes was generated and filtered to exclude all SNPs from phage regions or repetitive

sequences in the genome reference CT18 as defined previously (Table S2) [62]. Briefly, SNPs were filtered from excluded regions totalling 346,834 bases from an alignment of 43,100 SNPs using the python script embedded in the RedDog pipeline. Gubbins (v2.3.2) [66] was used to filter out additional SNPs in recombinant regions. Finally, the alignment of 16,324 SNPs from mapping of the remaining 462 isolates was utilized for phylogenetic analysis of the UKHSA dataset (Figure 1) and an alignment of 2,118 SNPs was used to construct phylogenetic analysis for H58 and nearest neighbours (Figure 2). Resultant BAM files for all isolates from RedDog mapping were used to determine previously defined genotypes according to an extended genotyping framework using the GenoTyphi pipeline [32] (available: https://github.com/katholt/genotyphi).

Phylogenetic analysis
RAxML (v8.2.9) [67] was used to infer maximum likelihood (ML) phylogenetic trees from the final chromosomal SNP alignment, with a generalized time-reversible model, a gamma distribution to model site-specific rate variation (the GTR+ Γ substitution model; GTRGAMMA in RAxML), and 100 bootstrap pseudo-replicates to assess branch support. Salmonella Paratyphi A AKU1_12601 (accession no: FM200053) [68] was used as an outgroup. The resultant trees were visualized using Interactive Tree of Life (iTOL) [69] and the ggtree package in R [70]. An interactive visualisation of this phylogeny and associated metadata can be found in Microreact (https://microreact.org/project/hzELvWqY3UCvsyAw892fnd-origins-of-h58-s-typhi) [71].

Characterisation of AMR associated genes and mobile elements
SRST2 (v0.2.0) [72] was used to detect AMR genes and plasmid replicons using the ARGannot [73] and PlasmidFinder [74] databases, respectively. Mutations in the gyrA and parC genes, as well as the R717Q mutation in acrB, were detected using Mykrobe v0.10.0 [75].
Raw read data for all S. Typhi sequences included in the chromosomal SNP analysis described above were mapped to the reference sequence of IncHI1 plasmid pAKU_1 (accession number AM412236) using RedDog (as described above). Those plasmid sequences where a read depth of at least 10-fold and coverage across the reference sequence of at least 75 % were observed were included in SNP analysis, with repetitive regions excluded with Gubbins (v.2.3.2) [66]. An alignment of 15 SNPs was used as input for plasmid haplotype assignment, which was carried out manually in R (v4.1.2) using the package ape (v5.7.1). SNP distances were determined using snp-dists (v0.7.0). Minimum spanning trees were inferred and visualised using the MSTree method within GrapeTree (v. 1.5.0) [76].

Bayesian phylogenetic analysis of H58 and nearest neighbours
Our estimation of the temporal signal of our H58 and nearest neighbour data exhibited a strong correlation between the sampling dates and the root-to-tip distances, with a positive value for the slope and an R2 value of 0.4743 (Supplementary figure 3). Additionally, the randomly reassigned sampling time of sequences 20 times to generate the mean rates indicated that there was no overlap between the 95% credible intervals of the mean rate of the real data set and that of the date randomization data (Supplementary figure 4). To infer where and when the first H58 (genotype 4.3.1) organism emerged, we conducted Bayesian phylogenetic analyses on a subset (n=345) of H58 (genotype 4.3.1) from our dataset and from published literature isolated between 1980 and 2000 [14, 30, 33]. This analysis of 345 S. Typhi isolates was conducted in the BEAST v1.8.4 [35]. The temporal signal of the data was checked initially. The maximum likelihood tree, constructed using the GTR+ Γ substitution model and GTRGAMMA, was subjected to TempEst v1.5 to test the best fit of linear regression between sampling dates and their root-to-tip genetic distances, using default TempEst parameters [77]. To further test temporal signal, the TipDatingBeast R package was used to randomly reassign the sampling dates of sequences 20 times to create date-randomized data sets. BEAST analyses were conducted for these randomized data sets and the mean rates were compared between runs. The data had sufficient temporal signal if the 95% credible interval of mean rates of the date-randomized datasets did not overlap with that of the original sampling dataset [78, 79].
An automatic model selection program (ModelFinder) [80] was implemented through IQ-TREE [81] and run on the non-recombinant SNP alignment (724 variable sites) to select the best-fit sequence evolution model for BEAST analysis. ModelFinder showed that GTR had the lowest Bayesian Information Criteria (BIC) score and thus it was chosen as the best-fit substitution model.
As part of the BEAST analysis, six different model combinations were run for six combinations, and the final analysis was conducted using the best fitting model. The path sampling and stepping-stone sampling approaches were applied to compare the log marginal likelihoods of the different runs [36, 82]. The GTR+Γ4 with strict clock and Bayesian skyline was identified as the best-fit model for running BEAST. Finally, BEAST was run three independent times using the best-fit model, using a Bayesian Markov Chain Monte Carlo (MCMC) parameter-fitting approach (generated 107 chains and sampled every 1000 iterations). The log files after three runs were combined using LogCombiner v1.8.3 [83] with a burn-in rate of 10%. The effective sample size (ESS) of all parameters was assessed by Tracer v1.8.3 [84]. If the ESS of any parameters was less than 200, we increased the MCMC chain length by 50% and reduced the sampling frequency accordingly [36]. The trees were combined and summarized using LogCombiner v1.8.3 and TreeAnnotator v1.8.3 [35].

For manuscripts utilizing custom algorithms or software that are central to the research but not yet described in published literature, software must be made available to editors and reviewers. We strongly encourage code deposition in a community repository (e.g. GitHub). See the Nature Portfolio guidelines for submitting code & software for further information.

Data

Policy information about availability of data

All manuscripts must include a data availability statement. This statement should provide the following information, where applicable:

- Accession codes, unique identifiers, or web links for publicly available datasets
- A description of any restrictions on data availability
- For clinical datasets or third party data, please ensure that the statement adheres to our policy

Raw reads were deposited in the European Nucleotide Archive (ENA) under study accession number PRJEB15284 (Table S1).

Research involving human participants, their data, or biological material

Policy information about studies with human participants or human data. See also policy information about sex, gender (identity/presentation), and sexual orientation and race, ethnicity and racism.

Reporting on sex and gender	n/a (not included)
Reporting on race, ethnicity, or	n/a (not included)

other socially relevant groupings	
Population characteristics	Data from United Kingdom Health Security Agency (UKHSA, formerly Public Health England) containing information on archived S. Typhi organisms isolated between 1980 and 1995 from travellers returning to the UK from overseas and receiving a blood culture were analysed.<br><br>The database was queried and organisms were selected from the following three categories: i) 126 S. Typhi with the E1 Phage type, which was thought to be associated with H58 [12], originating from South Asia (India, Nepal, Pakistan, and Bangladesh), ii) 159 S. Typhi organisms with a variety of non-E1 phage types originating from South Asia, and iii) 184 S. Typhi organisms with a variety of phage types (both E1 and non-E1) originating from locations outside of South Asia. A total of 470 S. Typhi organisms (out of 3,751 total S. Typhi isolated from returning travellers by UKHSA between 1980 and 1995) meeting these criteria were randomly selected, revived, subjected to DNA extraction and whole genome sequenced. Ultimately, our dataset was composed of 463 novel sequences generated as a component of this study and 305 existing sequences known to belong to the H58 lineage and its nearest neighbours, yielding a total of 768 whole genome sequences on which to structure subsequent analysis.
Recruitment	n/a, use of patient deidentified retrospective data
Ethics oversight	n/a, use of patient deidentified retrospective data

Note that full information on the approval of the study protocol must also be provided in the manuscript.

Field-specific reporting

Please select the one below that is the best fit for your research. If you are not sure, read the appropriate sections before making your selection.

☒ Life sciences ☐ Behavioural & social sciences ☐ Ecological, evolutionary & environmental sciences

For a reference copy of the document with all sections, see nature.com/documents/nr-reporting-summary-flat.pdf

Life sciences study design

All studies must disclose on these points even when the disclosure is negative.

Sample size	retrospective opportunistic analysis (n/a)
Data exclusions	any samples that could not be revived, or sequences of inferior coverage/quality, were excluded
Replication	phylogenetic analyses could be replicated using the same analytic pipelines and publicly available sequence and assembly data
Randomization	n/a
Blinding	n/a

Reporting for specific materials, systems and methods

We require information from authors about some types of materials, experimental systems and methods used in many studies. Here, indicate whether each material, system or method listed is relevant to your study. If you are not sure if a list item applies to your research, read the appropriate section before selecting a response.

Materials & experimental systems

n/a	Involved in the study	
☒	☐	Antibodies
☒	☐	Eukaryotic cell lines
☒	☐	Palaeontology and archaeology
☒	☐	Animals and other organisms
☒	☐	Clinical data
☒	☐	Dual use research of concern
☒	☐	Plants

Methods

n/a	Involved in the study	
☒	☐	ChIP-seq
☒	☐	Flow cytometry
☒	☐	MRI-based neuroimaging

Plants

Seed stocks	n/a
Novel plant genotypes	n/a
Authentication	n/a
